# Knowledge mapping of copper-induced cell death: A bibliometric study from 2012 to 2022

**DOI:** 10.1097/MD.0000000000031133

**Published:** 2022-11-11

**Authors:** Xue Ren, Ciming Pan, Zimeng Pan, Shanshan Zhao, Chen Wu, Wan Chen, Mengchen Liu, Xingyue Han, Hongying Kuang, Miao Qu

**Affiliations:** a Hei Longjiang University of Chinese Medicine, Hei Longjiang, China; b Yunnan University of Chinese Medicine, Yunnan, China; c First Affiliated Hospital, Heilongjiang University of Chinese Medicine, Hei Longjiang, China.

**Keywords:** bibliometric analysis, *Cite Space*, copper-induced cell death, visualization, web of science

## Abstract

The recent article *Copper induces cell death by targeting lipoylated TCA cycle proteins* has attracted much attention. Although copper-induced cell death has only recently been formally proposed, it has been studied much earlier. This study aims to undertake a bibliometric analysis of the literature on copper-induced cell death to understand the development of copper-induced cell death better and identify potential new research directions.

**Methods:**

With the help of *Cite Space* software, visual analysis is carried out on the annual number of published papers, countries/regions and institutions, journals co-citation, literature co-citation and reference burst, keywords co-occurrence, clustering, and burst.

**Results:**

A search of 770 articles published in English over the last ten years showed a fluctuating trend of increasing numbers of articles. China had the highest number of articles (190% or 24.68%), followed by the USA and India. Inflammation, biological evaluation, nanoparticle, and cu(ii) have been popular research themes in the last 4 years. The keyword clusters are summarized in 8 categories, including exposure, complexe, er stress, cleavage, paraptosis, cancer, glutamate, reactive oxygen species (ROS), expression. The hot topics are mainly focused on the exploration of mechanisms and related diseases, including induced apoptosis, aggregation, autophagy, endoplasmic reticulum stress, induced oxidative stress, and inflammation. Parkinson’s disease and cancer are 2 diseases that are closely related to copper-induced cell death.

**Conclusion:**

This study provides a visual analysis of copper-induced cell death trends and provides some hidden potentially useful information for future research directions.

## 1. Introduction

As one of the redox-active metal ions essential for human survival, copper maintains a dynamic equilibrium in the human body. Environmental factors can induce abnormal copper homeostasis, which is copper deficiency or copper overload, and imbalance in copper homeostasis can induce abnormal cellular autophagy, cytotoxicity, etc.^[1]^ Recently, the team of Peter Tsvetkov and Todd R. Golub at the Broad Institute of MIT and Harvard University has proposed for the first time a novel mode of cell death, copper-induced cell death,^[2]^ which occurs mainly by the mechanism of excess copper ions The main mechanism for this is that excess copper ions bind to thioredoxin in the tricarboxylic acid cycle pathway, resulting in abnormal levels of thioredoxin and loss of iron-sulfur cluster proteins, thereby triggering a proteotoxic stress response and ultimately causing cell death.^[3]^ Evidence suggests that although copper-induced cell death is distinct from the known regulated cell death (RCD) modalities such as apoptosis, autophagy, cell scorching and iron death, it still shares common hallmarks and characteristics of different forms of RCD, such as induction of reactive oxygen species (ROS) production, inhibition of the ubiquitin-proteasome system The RCD is a common marker and characteristic of different forms of RCD, such as induction of ROS, inhibition of the ubiquitin-proteasome system, etc.^[4]^ Because copper is involved in a variety of biological activities in the body, including oxidative stress, lipid metabolism and neurotransmitter synthesis,^[5]^ imbalances in copper homeostasis are closely associated with the development of many diseases, including neurodegenerative disorders, diabetes, and tumors.^[6]^ Wilson‘s disease, for example, is caused by copper overload due to ATP7B deficiency, which induces other system dysfunctions such as liver and nervous system.^[7]^ α-Ssynuclein aggregation is thought to be an important participant in the pathogenesis of Parkinson’s disease, so it has been proposed that when copper is overloaded, it causes neuronal cell death and α-synuclein aggregation, inducing Parkinson’s disease.^[8]^ Besides, Zn deficiency and simultaneous increase in Cu levels is associated with elevated levels of oxidative stress, which may exacerbate diabetic microangiopathy. While increased levels of free Cu ions may be associated with glycosylation and the release of Cu ions from the Cu-binding sites of proteins, so the application of Zn supplements and selective Cu chelators may help to alleviate oxidative stress and prevent further exacerbation of diabetic retinopathy.^[3]^ Cancer, a difficult disease for current medicine to overcome, has been a hot topic in the field of research. Recently, it has been found that elevated copper levels are closely associated with the development of several malignant tumors.^[9]^ Therefore, adjustment of copper homeostasis has also become one of the strategies to combat tumors. Currently, there are 2 main mechanisms, one is to use copper complexes to reduce copper ion content and inhibit tumor cell proliferation; the other is to transfer copper ions from extracellular to intracellular via its carrier, which increases intracellular Cu^2 + ^levels and induces oxidative stress, resulting in tumor cell death.^[4]^ Thus, the use of copper-induced cell death as a target or the use of copper therapy is expected to be a new way of cancer treatment. The mechanism of copper metabolism and copper-induced cell death is still unclear. Therefore, it is of great significance and prospect to further study the mechanism of copper metabolism homeostasis and related signaling pathways. The application of copper chelators is expected to become an important part of adjuvant therapy for a variety of diseases.

As the concept of copper-induced cell death has just been introduced, researchers are still in an imperfect state in terms of previous research in the field, so it is a major challenge for researchers to effectively sort through the past literature and further explore the deeper research directions in the field. As a discipline that efficiently counts the current status and evolution of the literature in the field and predicts cutting-edge trends, bibliometrics can effectively capture the overall trends in the field and provide insights into the hotspots of research, thus showing researchers the latest research trends.^[10]^ Many researchers have used bibliometrics to explore the content of research in this field. To date, no bibliometrics study on copper induced-cell death has been conducted, so this study aims to evaluate the literature on copper-induced cell death in the last decade using *Cite Space* software. The analysis will be carried out in 5 areas: annual publications, countries/regions and institutions, co-cited journals, literature co-citation and literature emergence, and keywords, in order to describe the current status of research in the field and outline the trends in the field, with the hope of providing a reference for researchers in the field to choose their research direction and an objective basis for further research in the field.

## 2. Materials and methods

### 2.1. Data source

**Figure 1.**
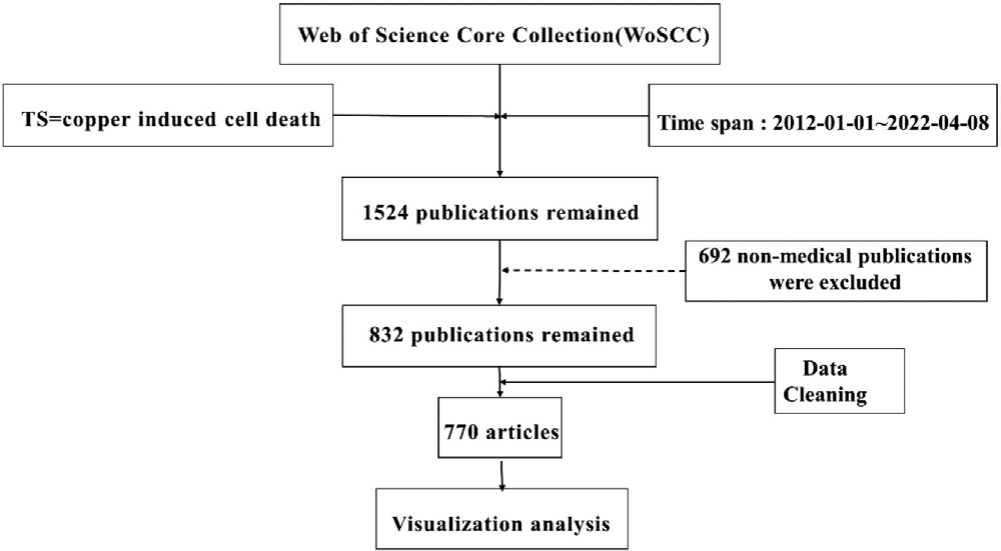
Retrieval flowchart.

The data for this study were obtained from the Web of Science Core Collection (WoSCC) with the search term set to TS = copper-induced cell death. The Time-slicing was set from January 1st, 2012 to April 8th, 2022. The language was limited to English. The limitation of literature type only included article. After excluding non-medical subjects such as fisheries and water resources, the remaining 832 articles were de-duplicated and excluded, resulting in the inclusion of 770 articles. All data in the text were extracted on the same day, April 8th, 2022, to avoid bias due to daily updates of the database. Each data entry was downloaded as a complete record in plain text format. The flowchart of the search is shown (Fig. 1). The data in this article are from WOS and are shared data, so the approval of the ethics committee is not required.

### 2.2. Data processing and analysis

The data was imported into Microsoft Excel 2019 and *Cite Space* 5.8.R3 for further analysis, generating visualization maps finally. Based on the results of the visual analysis, the filtered literature will be analyzed in terms of annual publication volume, country/region and institution, author co-citation, journal co-citation, Co-Cited Reference and Reference Burst, keyword co-occurrence, clustering, and burst detection.

*Cite Space* was set up as follows: time slices were chosen from 2012 to 2021, 1 year per slice (1); criteria were chosen (g-index, *k* = 14); term sources and node types were set up with flexible parameters according to specific needs.

## 3. Results

### 3.1. Annual growth trend of publications

**Figure 2.**
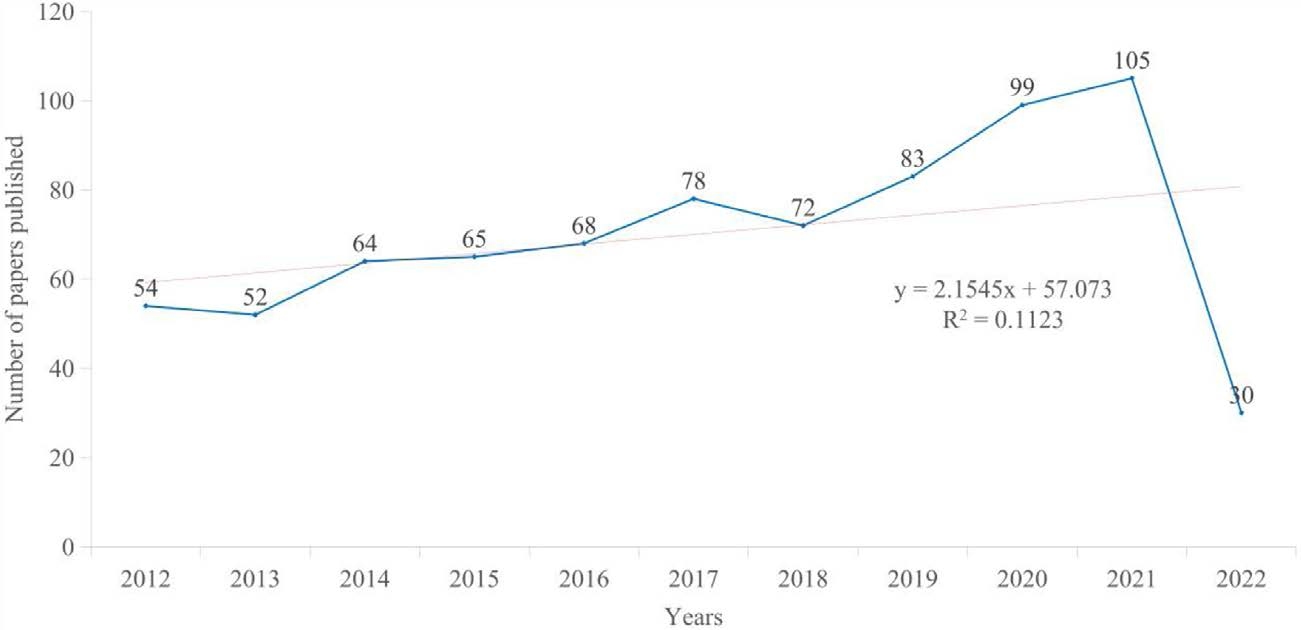
Annual output of copper-induced cell death research.

A search of the subject terms yielded 770 relevant publications. The volume of published literature related to copper-induced cell death has been increasing year by year (Fig. 2). The trend of the number of publications on copper-induced cell death can be divided into 2 phases.

### 3.2. Countries/regions and institutions analysis

**Figure 3.**
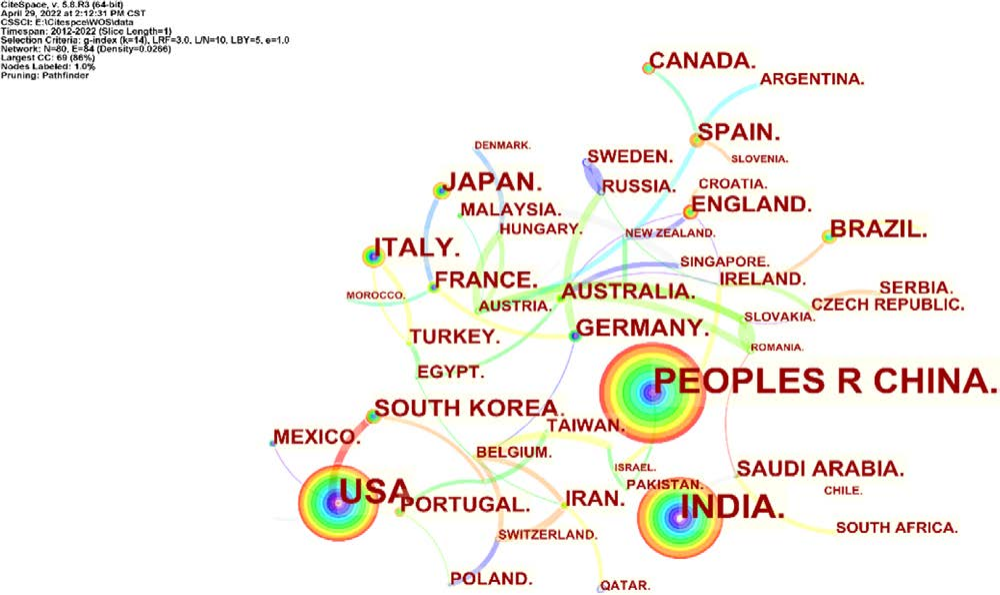
CiteSpace network visualization map of country/regions involved in copper-induced cell death research.

**Figure 4.**
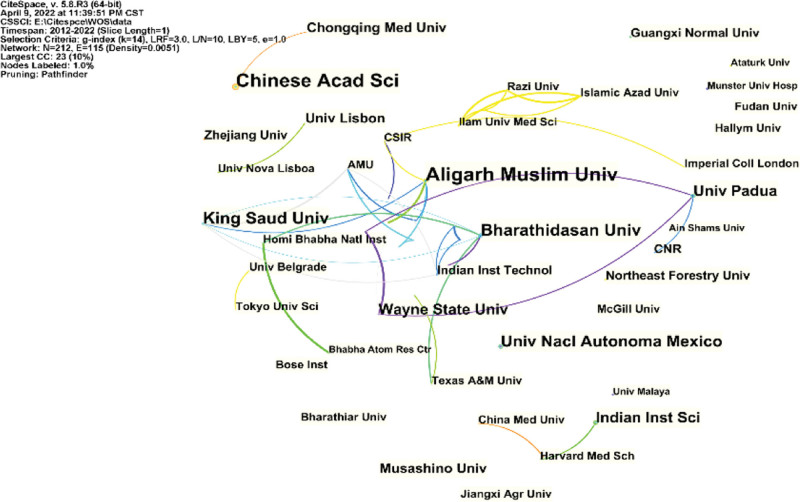
CiteSpace network visualization map of institutions involved in copper-induced cell death research.

The results of this analysis cover 212 institutions in 80 countries/regions. As shown in Figure 3, although the degree of centrality of each country is limited, there is extensive cooperation between countries in this field of study, reflecting a global trend of development and cooperation. The ten countries with the most research results in this field are shown (Table 1), with China in the first place (190 articles, 16.82%), followed by the USA (162 articles, 14.34%). Figure 4 shows the research of the institutions within the field, similar to the situation of Countries/Regions.

**Table 1:** Top 10 productive country related to copper-induced cell death research.

Rank	Countries	Articles	Percentage (N/770)
1	China	190	24.68%
2	United States	162	21.03%
3	India	143	18.57%
4	Italy	48	6.23%
5	Japan	41	5.32%
6	South Korea	32	4.15%
7	Spain	30	3.90%
8	Germany	30	3.90%
9	Brazil	28	3.64%
10	England	25	3.25%

### 3.3. Analysis of co-cited academic journals

Out of the 337 co-cited journals, 13 journals have more than 200 citations. As the authors can see from Table 2, the *Journal of Biological Chemistry* has the most citations, followed by the *Academy of Sciences of The United States of America, Plos One*, and *Free Radical Biology & Medicine*.

**Table 2:** Top 10 productive country related to copper-induced cell death research.

Rank	Co-cited journal	IF (2020)	JCR division	Country
1	Journal of Biological Chemistry	5.157	Q2	USA
2	Proceedings of the National Academy of Sciences of the United States of America	11.205	Q1	USA
3	PLoS One	3.24	Q2	USA
4	Free Radical Biology and Medicine	7.376	Q1	USA
5	Science	47.728	Q1	USA
6	Journal of Inorganic Biochemistry	4.155	Q2	USA
7	Nature	49.962	Q1	England
8	Cancer Research	12.701	Q1	USA
9	Journal of the American Chemical Society	15.419	Q1	USA
10	Chemical Reviews	60.622	Q1	USA

### 3.4. Co-cited reference and reference burst

**Figure 5.**
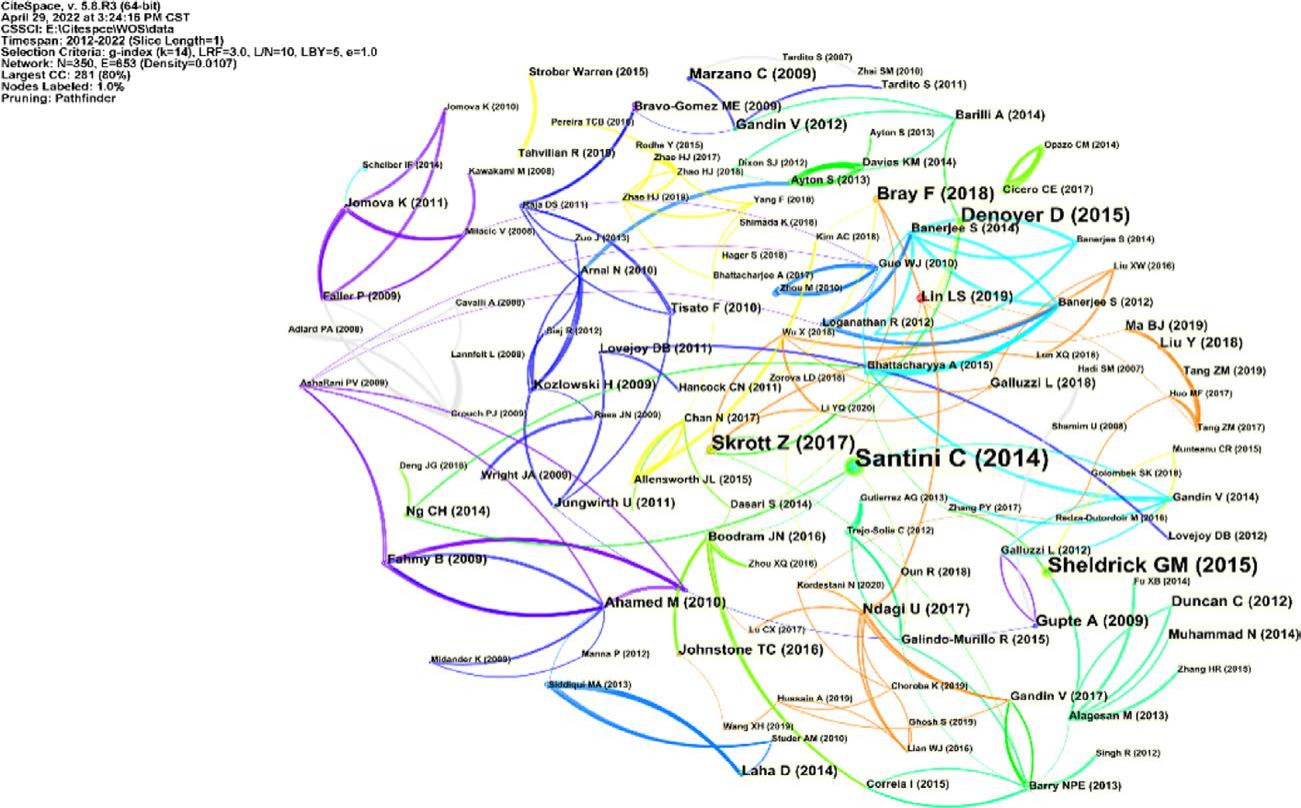
CiteSpace network visualization map of co-cited reference in copper-induced cell death research.

As shown in Table 3, the most cited reference is *Advances in copper complexes as anticancer agents* published in *Chemical Reviews* by Santini C et al in 2014, which demonstrates the fundamental status of their research in the field of copper-induced cell death. As shown in Figure 5 and Table 4, the authors detected the 25 references with the strongest citation bursts 16% (4/25) of the references showed citation bursts in 2017.

**Table 3:** Top 10 co-cited references in copper-induced cell death research.

Rank	Co-citation	Centrality	Author	Title
1	48	0	Santini C	Advances in copper complexes as anticancer agents
2	20	0	Denoyer D	Targeting copper in cancer therapy: “Copper That Cancer”
3	19	0	Skrott Z	Alcohol-abuse drug disulfiram targets cancer via p97 segregate adaptor NPL4
4	14	0	Bray F	Global cancer statistics 2018: GLOBOCAN estimates of incidence and mortality worldwide for 36 cancers in 185 countries
5	9	0	Gupte A	Elevated copper and oxidative stress in cancer cells as a target for cancer treatment
6	8	0	Marzano C	Copper complexes as anticancer agents
7	8	0	Duncan C	Copper complexes as therapeutic agents
8	8	0	Ndagi U	Metal complexes in cancer therapy – an update from drug design perspective
9	7	0	Laha D	Interplay between autophagy and apoptosis mediated by copper oxide nanoparticles in human breast cancer cells MCF7
10	7	0	Ahamed M	Genotoxic potential of copper oxide nanoparticles in human lung epithelial cells

**Table 4:** Top 25 references with the strongest citation bursts.

References	Year	Strength	Begin	End	2012–2022
Gupte A, 2009, Cancer Treat Rev, V35, P32, DOI 10.1016/j.ctrv.2008.07.004, DOI	2009	4.03	**2012**	2014	▃▃▃▂▂▂▂▂▂▂▂
Marzano C, 2009, Anti-Cancer Agent ME, V9, P185, DOI 10.2174/187152009787313837, DOI	2009	3.58	**2012**	2014	▃▃▃▂▂▂▂▂▂▂▂
Kozlowski H, 2009, Coordin Chem Rev, V253, P2665, DOI 10.1016/j.ccr.2009.05.011, DOI	2009	2.68	**2012**	2014	▃▃▃▂▂▂▂▂▂▂▂
Ahamed M, 2010, Biochem Bioph Res CO, V396, P578, DOI 10.1016/j.bbrc.2010.04.156, DOI	2010	3.23	**2013**	2015	▂▃▃▃▂▂▂▂▂▂▂
Fahmy B, 2009, Toxicol In Vitro, V23, P1365, DOI 10.1016/j.tiv.2009.08.005, DOI	2009	3.14	**2013**	2014	▂▃▃▂▂▂▂▂▂▂▂
Bravo-Gomez ME, 2009, J Inorg Biochem, V103, P299, DOI 10.1016/j.jinorgbio.2008.10.006, DOI	2009	2.61	**2013**	2014	▂▃▃▂▂▂▂▂▂▂▂
Jungwirth U, 2011, Antioxid Redox Sign, V15, P1085, DOI 10.1089/ars.2010.3663, DOI	2011	3.15	**2014**	2015	▂▂▃▃▂▂▂▂▂▂▂
Lovejoy DB, 2011, Cancer Res, V71, P5871, DOI 10.1158/0008-5472.CAN-11-1218, DOI	2011	2.93	**2014**	2014	▂▂▃▂▂▂▂▂▂▂▂
Tisato F, 2010, Med Res Rev, V30, P708, DOI 10.1002/med.20174, DOI	2010	2.93	**2014**	2014	▂▂▃▂▂▂▂▂▂▂▂
Duncan C, 2012, Metallomics, V4, P127, DOI 10.1039/c2mt00174h, DOI	2012	3.58	**2015**	2017	▂▂▂▃▃▃▂▂▂▂▂
Banerjee S, 2014, Chem Commun, V50, P5590, DOI 10.1039/c4cc02093f, DOI	2014	2.76	**2015**	2016	▂▂▂▃▃▂▂▂▂▂▂
Santini C, 2014, Chem Rev, V114, P815, DOI 10.1021/cr400135x, DOI	2014	15.57	**2016**	2019	▂▂▂▂▃▃▃▃▂▂▂
Sheldrick GM, 2015, Acta Crystallogr C, V71, P3, DOI 10.1107/S2053229614024218, DOI	2015	6.82	**2017**	2020	▂▂▂▂▂▃▃▃▃▂▂
Denoyer D, 2015, Metallomics, V7, P1459, DOI 10.1039/c5mt00149h, DOI	2015	5.42	**2017**	2020	▂▂▂▂▂▃▃▃▃▂▂
Muhammad N, 2014, Curr Opin Chem Biol, V19, P144, DOI 10.1016/j.cbpa.2014.02.003, DOI	2014	3.56	**2017**	2017	▂▂▂▂▂▃▂▂▂▂▂
Barilli A, 2014, Mol Pharmaceut, V11, P1151, DOI 10.1021/mp400592n, DOI	2014	2.48	**2017**	2018	▂▂▂▂▂▃▃▂▂▂▂
Skrott Z, 2017, Nature, V552, P194, DOI 10.1038/nature25016, DOI	2017	4.9	**2018**	2022	▂▂▂▂▂▂▃▃▃▃▃
Ng CH, 2014, Metallomics, V6, P892, DOI 10.1039/c3mt00276d, DOI	2014	2.98	**2018**	2019	▂▂▂▂▂▂▃▃▂▂▂
Liu Y, 2018, ACS Nano, V12, P4886, DOI 10.1021/acsnano.8b01893, DOI	2018	2.46	**2019**	2022	▂▂▂▂▂▂▂▃▃▃▃

### 3.5. Analysis of keywords

#### 3.5.1. Cooccurrence analysis.

**Figure 6.**
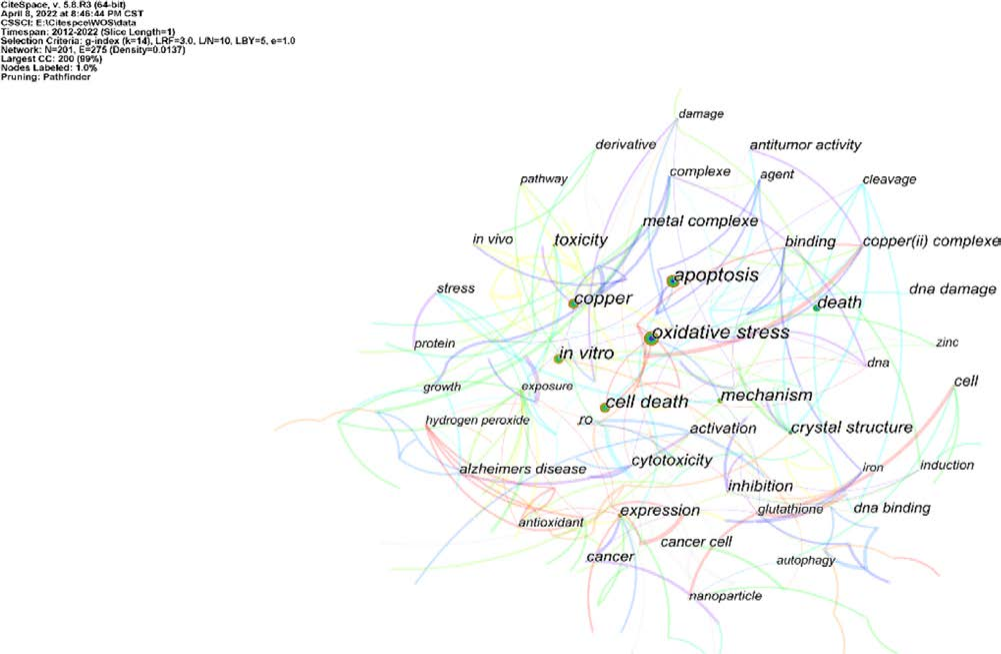
Keywords cooccurrence network in copper-induced cell death research.

The keyword co-occurrence graph for copper-induced cell death is shown in Figure 6 and Table 5. The density value was 0.0137. The ten most frequent keywords were oxidative stress, apoptosis, cell death, copper, in vitro, mechanism, death, toxicity, crystal structure, and cytotoxicity. Therefore, the authors have reason to believe that the relevant mechanisms of copper-induced cell death include oxidative stress, apoptosis, and cytotoxicity. In addition, copper-induced cell death is also involved in the development of cancer and Alzheimer’s disease.

**Table 5:** Top 10 keywords cooccurrence in copper-induced cell death research.

Rank	Count	Centrality	Year	Keyword
1	158	0.08	2012	Oxidative stress
2	136	0.11	2012	Apoptosis
3	117	0.02	2012	Cell death
4	101	0.04	2012	Copper
5	93	0.07	2012	In vitro
6	76	0.25	2012	Mechanism
7	74	0.04	2012	Death
8	62	0.03	2012	Toxicity
9	61	0.27	2012	Crystal structure
10	59	0.13	2012	Cytotoxicity
11	57	0.04	2012	Inhibition
12	55	0.07	2013	Metal complexes
13	54	0.21	2012	Expression
14	49	0.08	2012	Activation
15	48	0.08	2012	Cell
16	45	0.09	2013	Copper(ii) complexes
17	45	0.3	2012	Binding
18	44	0.15	2012	Cancer
19	42	0	2012	DNA damage
20	41	0.15	2012	RO
21	40	0.02	2012	Cancer cell
22	36	0.03	2012	Alzheimer’s disease
23	35	0.02	2012	Antitumor activity
24	34	0.02	2013	DNA binding
25	30	0.04	2012	Derivative
26	30	0.19	2013	Stress
27	28	0.26	2012	Complexes
28	28	0.01	2012	In vivo
29	27	0.17	2013	Cleavage
30	27	0.08	2014	Agent

#### 3.5.2. Cluster analysis.

**Figure 7.**
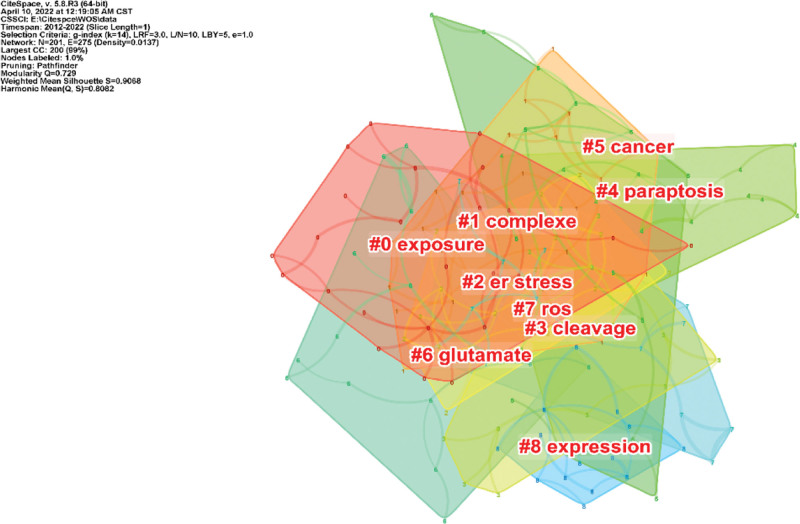
Keywords clusters in copper-induced cell death research.

The clustering analysis of the keywords yielded 201 nodes and 275 links, with a Q value of 0.729 (>0.3) and an S value of 0.9066 (>0.5), indicating a beneficial clustering effect, resulting in a total of 8 clusters, including: exposure, complexe, er stress, cleavage, paraptosis, cancer, glutamate, ROS, expression (Fig. 7 and Table 6). Cluster #0 is the largest cluster with 5 keywords: exposure, toxicity, heavy metal, zebrafish, drug discovery. The topic of cluster #1 includes 5 keywords, namely complexe, zinc, apoptosis inducer, membrane, and pyroptosis. The topic of cluster #2 includes 5 keywords, namely er stress, ROS, cells in vitro, neurodegenerative malady, and acute lymphoblastic leukemia. The topic of cluster #3 includes 5 keywords, namely cleavage, crystal structure, DNA binding, metal complexe, and copper(ii) complexes. Cluster #4 focuses on cell death and includes 6 keywords: paraptosis, oxidative stress, in vitro, copper, and photothermal therapy. Cluster #5 focuses on the cancer cell and includes 5 keywords: cancer, disulfiram, LC3, proteasome inhibitors, and prooxidant. The topic of cluster #6 includes 5 keywords, namely glutamate, oxidative stress, mechanism, chelation therapy, and copper chaperone. Cluster #7 is related to the passways and includes 5 keywords: ROS, DNA breakage, NF kappa b, keap1, complex i. The last cluster mainly deals with cancer and includes 5 keywords: expression, schiff base, independent paraptosis, l-buthionine sulfoximine, and cancer stem cells.

**Table 6:** Top 9 keywords cluster in copper-induced cell death research.

Cluster ID	Size	Centrality	Year	Cluster label (LLR)
0	24	0.922	2016	Exposure (9.66, 0.005); toxicity (8.15, 0.005); heavy metal (7.01, 0.01); zebrafish (6.23, 0.05); drug discovery (6.03, 0.05)
1	24	0.851	2013	Complexe (12.53, 0.001); zinc (11.72, 0.001); apoptosis inducer (8.35, 0.005); membrane (8.35, 0.005); pyroptosis (8.35, 0.005)
2	16	0.914	2015	Er stress (8.92, 0.005); reactive oxygen species (ROS) (8.19, 0.005); cells in vitro (5.61, 0.05); neurodegenerative malady (5.61, 0.05); acute lymphoblastic leukemia (5.61, 0.05)
3	15	0.959	2015	Cleavage (29.1, 1.0E-4); crystal structure (14.1, 0.001); DNA binding (13.88, 0.001); metal complexe (13.88, 0.001); copper(ii) complexes (9.83, 0.005)
4	14	0.928	2017	Paraptosis (16.48, 1.0E-4); oxidative stress (10.82, 0.005); in vitro (10.29, 0.005); copper (9.98, 0.005); photothermal therapy (5.86, 0.05)
5	14	0.883	2015	Cancer (10.36, 0.005); disulfiram (9.83, 0.005); lc3 (8.68, 0.005); proteasome inhibitors (8.68, 0.005); prooxidant (8.68, 0.005)
6	14	0.985	2013	Glutamate (12.15, 0.001); oxidative stress (12.11, 0.001); mechanism (8.36, 0.005); chelation therapy (8.1, 0.005); copper chaperone (8.1, 0.005)
7	14	0.901	2013	ROS (24.64, 1.0E-4); DNA breakage (7.6, 0.01); NF kappa b (6, 0.05); keap1 (5.64, 0.05); complex i (5.64, 0.05)
8	14	0.787	2014	Expression (11.09, 0.001); schiff base (6.24, 0.05); independent paraptosis (5.04, 0.05); l-buthionine sulfoximine (5.04, 0.05); cancer stem cells (5.04, 0.05)

#### 3.5.3. Burst detection.

As shown in Table 7, the first 16 keywords were selected to discover the research hotspots about copper death. These hot subject terms include induced apoptosis, aggregation, Parkinson’s disease, induction, zinc, autophagy, *Escherichia coli*, endoplasmic reticulum stress, target, induced oxidative stress, anticancer, inflammation, biological evaluation, crystal, nanoparticle, cu(ii).

**Table 7:** Top 16 burst keywords in articles related to copper-induced cell death research.

Keywords	Year	Strength		Begin	End	2012–2022
Induced apoptosis	2012	3.16	2012		2015	▃▃▃▃▂▂▂▂▂▂▂
Aggregation	2012	2.98		2012	2014	▃▃▃▂▂▂▂▂▂▂▂
Parkinson’s disease	2012	3.83		2013	2017	▂▃▃▃▃▃▂▂▂▂▂
Induction	2012	3.16		2013	2014	▂▃▃▂▂▂▂▂▂▂▂
Zinc	2012	4.04		2016	2017	▂▂▂▂▃▃▂▂▂▂▂
Autophagy	2012	3.11		2017	2020	▂▂▂▂▂▃▃▃▃▂▂
*Escherichia coli*	2012	2.91		2017	2018	▂▂▂▂▂▃▃▂▂▂▂
Endoplasmic reticulum stress	2012	2.62		2017	2018	▂▂▂▂▂▃▃▂▂▂▂
Target	2012	2.5		2017	2019	▂▂▂▂▂▃▃▃▂▂▂
Induced oxidative stress	2012	3.38		2018	2019	▂▂▂▂▂▂▃▃▂▂▂
Anticancer	2012	4.21		2019	2022	▂▂▂▂▂▂▂▃▃▃▃
Inflammation	2012	3.31		2019	2022	▂▂▂▂▂▂▂▃▃▃▃
Biological evaluation	2012	3.06		2019	2022	▂▂▂▂▂▂▂▃▃▃▃
Crystal	2012	2.72		2019	2020	▂▂▂▂▂▂▂▃▃▂▂
Nanoparticle	2012	3.72		2020	2022	▂▂▂▂▂▂▂▂▃▃▃
CU(II)	2012	2.94		2020	2022	▂▂▂▂▂▂▂▂▃▃▃

## 4. Discussion

### 4.1. General information

This study searched the WoSCC database for literature on copper-induced cell death in the last decade, and the screening yielded 770 articles published by 212 institutions in 80 countries/regions, with a total of 350 co-citations. Out of the 80 countries/regions, only 3 countries have published more than 100 papers. Co-cited journals (Table 2) show the most significant number of co-cited references on the topic of copper-induced cell death, received the largest number of co-cited references, which is a journal in the field of biochemistry and molecular biology.

### 4.2. Knowledge base

Co-cited documents are documents that are frequently cited by other publications. A knowledge base is a collection of documents frequently referred by relevant research groups,^[11]^ which is not exactly the same as highly cited literature. The top 10 references co-cited in this field are presented in Table S3 through a bibliometric analysis of the literature related to copper-induced cell death.

In 2014, the first co-cited paper was published in *Chemical Reviews* by 6 authors, led by Santini C, as a collaborative review entitled *Advances in copper complexes as anticancer agents*, demonstrating its outstanding contribution to the study of copper-induced cell death. This paper describes a form of cell death that is distinct from apoptosis. A specific class of copper complexes, in particular the thioxotriazole copper(II) complex [Cu(L49)Cl2], are potent inhibitors of CT-L protease activity, which allowed scientists to explore a new mode of cell death.

The second co-cited paper was published by Denoyer D in *Metallomics* in 2015. This paper describes the involvement of copper in angiogenesis, cancer growth and metastasis, and the finding of elevated copper levels in malignant tumors, laying the theoretical foundation for the subsequent investigation of the involvement of copper overload in the pathogenesis of diseases such as cancer.^[12]^

The third co-cited paper was published by Skrott Z et al In 2017 in Nature, titled *Alcohol-abuse drug disulfiram targets cancer via p97 segregase adaptor NPL4*.^[13]^ The paper explored the anticancer potential of disulfiram (tetraethylthiuram disulfide, DSF) and NPL4 was identified as the molecular target of tumor inhibition, and found that the antitumor effects of DSF could be enhanced by the presence of Cu.

All in all, the top 10 co-cited references focused on reviews (7 reviews were published from 2009 to 2018), involving relevant mechanisms including Oxidative Stress, Autophagy, and genotoxic potential. The authors also published reviews on biomarkers such as p97 and NPL4, as well as diseases related to copper-induced cell death, such as cancer, Cardiovascular disease, and Huntington’s disease. These have provided a solid foundation for future research in the emerging field of copper-induced cell death.

### 4.3. Research hotspots

The burst detection results suggest hotspots of research in this field. The current analysis shows that the hotspots of research in this field focus on mechanisms, including induced apoptosis, aggregation, autophagy, endoplasmic reticulum stress, induced oxidative stress, and inflammation. Parkinson’s disease and cancer are 2 diseases that are closely related to copper-induced cell death. The literature on Parkinson’s disease has mostly explored its pathogenesis concerning copper-induced cell death, while the literature on cancer has explored chiefly the use of copper complexes in cancer therapy. It is noteworthy that in 2020, the fastest growth in the number of articles in this field may be related to the term nanoparticle, which was the most popular in that year. Inflammation, biological evaluation, nanoparticle, and cu(ii)) have become popular research topics in the last 4 years. This article may suggest that future research will continue to focus on improving the biological evaluation of tumors, thus broadening the ideas of tumor therapy in the form of copper complexes.

An analysis of the dynamics of this field with keyword co-occurrence analysis and burst word detection reveals scholars focused on the mechanism in the early stage, and gradually presented the trend of parallel research on the mechanism and clinical diseases in the late stage. Since the clinical application of cisplatin-based drugs has many limitations, and copper complexes can better avoid their shortcomings, researchers have carried out many studies on copper complexes, drawing on their previous experience in mechanistic research, which has laid a solid foundation for effectively solving clinical problems. It is evident that mechanistic studies have been the focus of research in the field of copper-induced cell death. The proliferation of clinical problems has led researchers to explore further the mechanisms associated with cancer, which is currently the focus of research in this field. In recent years, research on copper-induced cell death and human disease is still relatively scarce, probably because this field is still in its infancy.^[14]^ More studies have focused on the therapeutic application of copper complexes, suggesting that their primary mechanism of action is the induction of DNA damage. However, this anti-proliferative mechanism sometimes does not exert a selective cytotoxic effect on cancer cells, thus making searching for other intracellular targets problematic. Therefore, the deeper the exploration of the mechanism of copper-induced cell death, the more rational the design of therapeutic drugs for cancer and other diseases, and the better the role of copper complexes in overcoming drug resistance.

The 8 clusters of keywords presented in this paper illustrate 8 research frontiers in this field, which to some extent represent 8 significant aspects of research in it, mostly related to the mechanisms involved in cancer treatment. Copper is considered a limiting factor in cancer progression, including growth, angiogenesis, and metastasis. As a result, copper complexes are of great interest for cancer therapy, with mechanisms such as endoplasmic reticulum stress, DNA cleavage, paraptosis, glutamate, and ROS. Specifically, for example, a phosphine copper(I) complex, [Cu(thp)(4)][PF(6)] (CP), was found to induce endoplasmic reticulum stress by inhibiting 26S proteasome activities, a novel mechanism for CP-induced cancer cell death is revealed: a possible association with a signaling pathways involved in paraptosis.^[15]^ Furthermore, it has been shown that copper chelation has excellent potential for cancer therapy by causing DNA Cleavage and contributing to the production of ROS.^[16]^ In addition, Ammonia, which is produced by glutamate metabolism, also accelerates the proliferation of breast cancer cells.^[17]^ Although these articles do not directly mention that copper-induced cell death, most of them describe the mechanisms of cancer cell death without identifying markers of other cell death types.

To some extent, the references with the highest citation bursts are unique contributions to the field of study. After analysis, the literature with the strongest citation burst is also the first co-cited paper – *Advances in copper complexes as anticancer agents*, which is a seminal work in the study of copper-induced cell death. Although the present article does not explicitly propose the clear concept of copper-induced cell death, it provides a discriminatory meaning for future copper-induced cell death as no markers associated with apoptosis were found. Its influence on the field is evident as it continues into 2019. More importantly, of the top 25 references with the highest citation bursts (Fig. 7), 8 references are still in bursts. These 8 references represent the most recent themes in copper-induced cell death and therefore deserve further discussion.^[18]^

Ranking according to burstness strength, the first paper (strength = 4.9) was also the third co-cited paper, citing bursts lasting 5 years (2018–2022). The reference with the fourth-strongest citation burstness (strength = 5.63) was also the fourth co-cited paper. In the 2018 CA-A CANCER JOURNAL FOR CLINICIANS, the citation burst lasted for 3 years (The fifth reference (strength = 3.66). The fifth reference (strength = 3.66) was also the eighth co-cited paper,^[19]^ and the citation burst lasted for 3 years (2020–2022). These 3 articles were all published after 2016, which are representative articles that started the rapid growth stage in the number of papers published in this field.

The second study (strength = 5.42) was published by Liu Y et al^[20]^ in *ACS Nano* 2018, with a citation burst lasting 4 years (2019–2022), which found that therapeutic agents known as copper ferrite nanospheres can effectively weaken the hypoxic and antioxidant capacity of tumors, further exploring the relationship between copper-induced cell death and redox reactions in terms of the drug applications.

The reference with the third-strongest citation burstness (strength = 2.46) was published by Johnstone TC et al^[21]^ in *CHEMICAL REVIEWS* 2016, citing bursts lasting 4 years (2019–2022). This article proposes the existence of a nanoparticle delivery modality for cisplatin prodrug (cisplatin prodrug) that is effective in avoiding drug resistance as it does not reduce the expression of the copper transporter CTR1. This paper illustrates the mechanism of copper ion-related effects in drug application, and it lays the theoretical foundation for the study of the mechanism of copper-induced cell death.

## 5. Limitations

Compared to previous reviews, *Cite Space* is an efficient way of helping researchers better to understand the dynamics of a field of study, and it also can gain a more comprehensive understanding of the current research focus in order to identify future research directions with greater precision. Even so, there are still shortcomings. Firstly, the analysis of this paper only includes the WoSCC database, which is not comprehensive enough to capture the results of all the databases and may be biased. Secondly, only the analysis of the application software is only based on the Annual Growth Trend of Publications, Countries/Regions, Institutions, Co-Cited Academic Journals, Co-Cited Reference, Reference Burst, and Keywords, and it did not provide a comprehensive view of all the information available in the field of copper-induced cell death. Although a selective analysis could have highlighted the key points, some details were inevitably left out. Finally, the results obtained in this paper have been processed by *Cite Space* software with certain algorithms, which may bias some of the results.^[22]^

## 6. Conclusion

In summary, by reviewing previous works in the field and conducting Cite Space bibliometric analysis in this paper for the first time, the authors have gained a deeper understanding of the topic of copper-induced cell death.

Since copper-induced cell death is a recently discovered mode of cell death, the most frequently occurring keywords in the field of copper-induced cell death are mostly related to its mechanism, with the main aspects including oxidative stress, endoplasmic reticulum stress, and apoptosis. In addition, this paper also finds that the alternative application of copper chelators related to the mechanism of copper-induced cell death in cancer may be a new research trend. Compared with traditional reviews, the *Cite Space*-based bibliometric study provides a clearer picture of the development and core of research in the field of copper-induced cell death and provides an objective reference for researchers to choose the right research direction. It is hoped that the research results of this paper will be helpful for deepening the research in this field in the future.

## Acknowledgments

The authors are indebted to the Miss Zhang Yunqi who contributed her time, knowledge, and energy to developing this document.

## Author contributions

All authors have read and approved the manuscript, and ensure that this is the case.

**Conceptualization:** Xue Ren, Zimeng Pan.

**Data curation:** Xue Ren, Xingyue Han.

**Formal analysis:** Xue Ren, Miao Qu.

**Investigation:** Ciming Pan.

**Methodology:** Shanshan Zhao, Hongying Kuang.

**Software:** Chen Wu, Mengchen Liu.

**Supervision:** Wan Chen.
